# The association between alcohol consumption and blood lipids in Chinese children and adolescent: findings from the China Health and Nutrition Survey

**DOI:** 10.1186/s12887-024-04807-x

**Published:** 2024-05-09

**Authors:** Qingting Bu, Lingyan Fang, Bo Huang, Huijun Cai, Zhenyu Pan

**Affiliations:** 1 Department of Genetics, Northwest Women’s and Children’s Hospital, Xi’an, Shaanxi China; 2 Department of Medical Quality Control, Yantaishi Penglai Second People’s Hospital, Yantai, Shandong China; 3 Department of Clinical Laboratory, Xi’an Children’s Hospital, Xi’an, Shaanxi China; 4 Department of Pharmacy, Xi’an Children’s Hospital, Xi’an, Shaanxi China; 5 School of Public Health, Xi’an Jiaotong University Health Science Center, Xi’an, Shaanxi China

**Keywords:** Alcohol consumption, Drink, Children, Adolescents, Blood lipid

## Abstract

**Background:**

Alcohol consumption by children and adolescents is receiving increasing attention. It may cause dyslipidemia, a risk factor for cardiovascular disease. However, the association between alcohol consumption and blood lipids in children and adolescents is unclear, and so we aimed to characterize this association.

**Methods:**

Data from the China Health and Nutrition Survey were extracted from children and adolescents aged 7–18 years for whom information was available on alcohol consumption. The population was divided into drinking and nondrinking groups. The χ2, Student’s t, or Mann–Whitney U test was used to compare groups. Univariate and multivariate linear regression and propensity score matching (PSM) analysis were used to identify the association between alcohol consumption and blood lipids.

**Results:**

This study included 408 children and adolescents with 35 drinkers and 373 nondrinkers. The drinkers had significantly lower values of total cholesterol (TC) (3.8 mmol/L for nondrinkers versus 3.5 mmol/L for drinkers, *p* = 0.002) and high-density lipoprotein cholesterol (HDL-C) (1.3 mmol/L for nondrinkers versus 1.2 mmol/L for drinkers, *p* = 0.007), but not for low-density lipoprotein cholesterol (LDL-C) (2.1 mmol/L for nondrinkers versus 2.0 mmol/L for drinkers, *p* = 0.092) or triglyceride (TG) (0.9 mmol/L for nondrinkers versus 0.8 mmol/L for drinkers, *p* = 0.21). The univariate and multivariate analyses led to the same conclusions. After PSM there was still a significant negative association between alcohol consumption and TC or HDL-C.

**Conclusion:**

Alcohol consumption in children and adolescents exhibited significant negative associated with TC and HDL-C, but not with LDL-C or TG. These findings need to be confirmed in future prospective research, and the health effects of blood lipid changes caused by drinking in children and adolescents need to be clarified.

## Background

Alcohol consumption is a global public health problem and a major risk factor for cardiovascular disease (CVD) [1, 2]. Alcohol was found to be responsible for an estimated net CVD burden of 593,000 deaths (3.3% of all CVD deaths) and 13 million CVD disability adjusted life years (DALYs) (3.2% of all CVD DALYs) globally [2]. CVD was responsible for 19.8% and 9.8% of all alcohol-attributable deaths and DALYs lost, respectively [2].

As a CVD risk factor, dyslipidemia may play a role in the relationship between alcohol and CVDs. The dyslipidemia characterized by the increase of low density lipoprotein cholesterol (LDL-C) or total cholesterol (TC) is an important risk factor for CVD [3]. Other types of dyslipidemia, such as increased TG or decreased HDL-C, are also associated with increased risk of CVD [4–7]. Some studies have investigated the association between alcohol consumption and blood lipids in adults, but the conclusions were inconsistent. Some studies found a significant relationship between alcohol consumption and high density lipoprotein cholesterol (HDL-C) levels [8–10]. The risk of low HDL-C in males and females decreases as alcohol consumption increases [8]. A J-shaped correlation was found between the alcohol consumption and the risk of high triglyceride (TG) levels in males, but there was no significant association between alcohol consumption and high TG levels in females [8]. Mansour et al. found that drinking was not significantly correlated with TG, TC, or HDL-C [11].

Alcohol consumption among children and adolescents has become an important public health issue. In 2018, a report released by the World Health Organization (WHO) showed that 25.0% of adolescents aged 12–15 drank alcohol, and drinking behavior is showing a trend towards younger age groups worldwide [12]. The study from China shows that the alcohol consumption rate of school-age children can reach 11.2%, and in some special groups, it can reach 22.1% [13]. This may foresee a high burden of CVD due to alcohol consumption in the future. Due to the difficulty in observing CVD in childhood and adolescence, elucidating the association between alcohol consumption and blood lipids in children and adolescents can help explain its relationship with CVD. This is of great significance for public health education. However, few studies have investigated the relationship between alcohol consumption and blood lipids in children and adolescents. Only two previous studies that we know of, published in 1979 and 1981 [14, 15], addressed the relationship between alcohol consumption and blood lipids in adolescents. However, the conclusions of those studies were inconsistent, with one [15] finding a significant positive association between alcohol consumption and HDL-C in adolescents, and the other [14] finding no significant association between adolescent drinking and blood lipids (TC, TG, HDL-C, or LDL-C).

This study therefore explored the effect of alcohol consumption on blood lipids in children and adolescents by analyzing the data from the China Health and Nutrition Survey (CHNS).

## Methods

### Study design and data sources

This was a cross-sectional study designed based on a subset of the CHNS, which is an ongoing national household-based study employing a multistage random cluster sampling method in nine provinces in China (Guangxi, Guizhou, Heilongjiang, Henan, Hubei, Hunan, Jiangsu, Liaoning, and Shandong) with different geographics, economic development levels, and health indicators [16, 17]. The CHNS was conducted successively in 1989, 1991, 1993, 1997, 2000, 2004, 2006, 2009, 2011, and 2015. The individual response rates were high for each survey wave from 1989 to 2006, with an average of 88% [17]. The obtained biomarker data needed to be analyzed at part of the present study. The CHNS 2009 data were adopted because this was the only time when fasting blood data were available [18]. We collected data on children and adolescents aged 7–18 years for whom information on alcohol consumption was available in the CHNS 2009 database, because such information for those younger than 7 years was not included in the database. Previous studies and the official CHNS website (https://www.cpc.unc.edu/projects/china) include detailed descriptions of the survey procedures and the anthropometric and clinical measurement methods [19–21]. In brief, demographic data were collected using a standardized questionnaire face-to-face; standardized questionnaires of the CHNS 2009 database can be obtained on this website, https://www.cpc.unc.edu/projects/china/data/questionnaires; standard procedures for anthropometric measurements were followed by well-trained examiners; 12 mL of fasting blood was collected via venipuncture, and then immediately centrifuged, and the serum was tested for related measurements. All children and their parents provided written informed consent.

### Outcome variable

Serum TC was measured using the cholesterol oxidase–phenol and aminophenazone method; serum HDL-C and LDL-C were measured using the enzymatic method; serum TG was measured using the glycerol-3-phosphate oxidase–phenol and aminophenazone method; reagents were from Kyowa (Japan) and measure machine was Hitachi 7600 for blood lipids detection.

### Covariates

The independent variable is drinking alcohol, and the covariates include age, sex, nationality, residence, per-capita household income, body mass index z score (BMIz), blood pressure, 3-day average dietary value. Drinking alcohol, age, sex, nationality, residence, per-capita household income and 3-day average dietary value were collected by face-to-face visit interviews and information questionnaires. For these variables surveyed through questionnaires, the accuracy of the 3-day average dietary value needs to be considered due to its difficulty in measuring compared to other variables. Although CHNS did not disclose the data for the questionnaire validity, it ensures the accuracy of the 3-day average dietary value and other variables through quality control measures. The survey questionnaire and survey manual used are uniformly printed. The interviewers have been graduates of post-secondary schools and uniformly trained. All interviewers have been trained nutritionists who are professionally engaged in nutrition work and have survey experience. The 3-day average dietary value from 24 h recall has been compared with each individual’s average daily dietary intake calculated from the household survey. If significant differences are found, the household and the individual in question were revisited. Drinking alcohol is defined as drinking any alcoholic beverage within a year in CHNS. Height and weight were measured by using a standardized protocol from WHO [22]. Body mass index (BMI) is the square of height in meters divided by weight in kilograms. BMIz was calculated as (individual BMI value minus mean BMI value)/(standard deviation of BMI) based on the published data according to sex and age [23]. Blood pressure was measured with the subjects in a sitting position, using a appropriate size of cuff for the children’s arm circumference and a sphygmomanometer with a precision of 5 mm Hg. For 3-day average dietary value, a 24 h recall method for three consecutive days for each participant was used to record the dietary. Parents were asked to recall the food consumption of children younger than 12 years. Average Energy, carbohydrate, fat and protein intake were calculated according to the China Food Composition by China CDC National Institute for Nutrition and Health. Children and adolescents with censored data on the analyzed variables were excluded. Cities, suburban areas, towns and county capital cities were classified as the urban area and villages as the rural area.

### Statistical analysis

Categorical data were expressed as percentages and compared using χ2 tests. Shapiro–Wilk test was used to check normal distribution. Continuous data conforming to a normal distribution were expressed as mean ± standard-deviation values and compared using Student’s t-test, and nonnormal continuous data were expressed as median (25th to 75th percentile) values and compared using Mann–Whitney U tests. Univariate and multivariate linear regression analyses were applied to the association between alcohol consumption and blood lipids. A propensity score matching (PSM) (1:4) analysis was used to minimize the bias between children and adolescents who do and do not drink alcohol. The matched data were used to further compare the blood lipid levels between the two groups. All analyses were conducted using R software (version 3.4.3), and a two-tailed probability values of *p* < 0.05 was considered indicative of statistical significance.

## Results

### Baseline participant characteristics

This study included 408 children and adolescents aged 7–18 years. Table 1 lists their basic demographics, anthropometrics, and clinical characteristics according to their alcohol consumption status. In total, 35 children and adolescents drank alcohol and 373 did not. Children and adolescents who drank alcohol had significantly lower TC (3.8 mmol/L for nondrinkers versus 3.5 mmol/L for drinkers, *p* = 0.002) and HDL-C (1.3 mmol/L for nondrinkers versus 1.2 mmol/L for drinkers, *p* = 0.007) values, but not LDL-C (2.1 mmol/L for nondrinkers versus 2.0 mmol/L for drinkers, *p* = 0.092) or TG (0.9 mmol/L for nondrinkers versus 0.8 mmol/L for drinkers, *p* = 0.21) values (Table 1). Since the study was not a randomized controlled trial, there were inevitable significant differences in factors such as age, sex, blood pressure, and 3-day average dietary value between the two groups (nondrinkers versus drinkers) (Table 1).

**Table 1:** Characteristics of the participants according to their alcohol consumption status

	**Nondrinker**	**Drinker**	***P* value**
**Participants, n**	373	35	
**Age (years), median (25th-75th percentile)**	14.0 (13.0–16.0)	15.0 (13.0–17.0)	0.008
**Sex, n (%)**			0.008
Male	184 (49.3)	26 (74.3)	
Female	189 (50.7)	9 (25.7)	
**Han Nationality, n (%)**			0.351
Yes	313 (83.9)	32 (91.4)	
No	60 (16.1)	3 (8.6)	
**Residence, n (%)**			0.095
Urban	104 (29.9)	15 (42.9)	
Rural	269 (72.1)	20 (57.1)	
**Per capita household income (RMB), median (25th-75th percentile)**	5607.5 (3150.0–10500.0)	6650.0 (4252.5–11976.4)	0.194
**BMIz, median (25th-75th percentile)**	-0.3 (-0.7–0.5)	-0.2 (-0.8–0.4)	0.56
**Blood pressure (mmHg), median (25th-75th percentile)**			
Systolic blood pressure	103.3 (97.3–111.0)	109.3 (100.0–111.7)	0.038
Diastolic blood pressure	70.0 (61.0–76.0)	71.3 (67.3–78.3)	0.06
**3-day average dietary value, median (25th-75th percentile)**			
3-day average energy (kcal)	1786.2 (1472.6–2237.4)	2349.6 (1961.0–2529.2)	< 0.001
3-day average carbohydrate (g)	256.2 (207.0–324.2)	320.7 (246.6–369.6)	0.006
3-day average fat (g)	57.0 (40.1–80.7)	79.1 (51.7–99.0)	0.003
3-day average protein (g)	56.6 (43.2–72.8)	69.5 (55.7–84.4)	0.006
**Blood lipids (mmol/L), median (25th-75th percentile)**			
Total cholesterol	3.8 (3.4–4.3)	3.5 (3.1–3.8)	0.002
High density lipoprotein cholesterol	1.3 (1.1–1.5)	1.2 (1.0–1.4)	0.007
Low density lipoprotein cholesterol	2.1 (1.7–2.6)	2.0 (1.8–2.2)	0.092
Triglyceride	0.9 (0.6–1.3)	0.8 (0.6–1.1)	0.21

### Univariate and multivariate linear regression

Variables that were statistically significant in the univariate linear regression analysis were analyzed as covariates in the multivariate linear regression to adjust for the influence of confounding factors on the association between alcohol consumption and blood lipids. In univariate analysis, sex, nationality, per capita household income, BMIz and drinking were significantly associated with TC; per capita household income and drinking were significantly associated with HDL-C; sex, per capita household income and BMIz were significantly associated with LDL-C, nationality and BMIz were significantly associated with TG (Table 2). Regarding the association between alcohol consumption and blood lipids, as listed in Table 2, alcohol consumption had significant negative associations with TC (coefficient =  − 0.36 and *p* = 0.003 for univariate regression, − 0.33 and *p* = 0.006 for multivariate regression) and HDL-C (coefficient =  − 0.15 and *p* = 0.049 for univariate regression, coefficient =  − 0.16 and *p* = 0.04 for multivariate regression) in both the univariate and multivariate regression analyses, but not with LDL-C (coefficient =  − 0.23 and *p* = 0.058 for univariate regression, − 0.19 and *p* = 0.098 for multivariate regression) or TG (coefficient =  − 0.15 and *p* = 0.173 for univariate regression, − 0.13 and *p* = 0.232 for multivariate regression).

**Table 2:** Univariate and multivariate linear regression analysis

	**TC as dependent variable**		**HDL-C as dependent variable**		**LDL-C as dependent variable**		**TG as dependent variable**	
	**Univariate regression** **Coefficient (*P* value)**	**Multivariate regression** **Coefficient (*P* value)**	**Univariate regression** **Coefficient (*P* value)**	**Multivariate regression** **Coefficient (*P* value)**	**Univariate regression** **Coefficient (*P* value)**	**Multivariate regression** **Coefficient (*P* value)**	**Univariate regression** **Coefficient (*P* value)**	**Multivariate regression** **Coefficient (*P* value)**
**Age**	0.02 (0.258)		0.01 (0.4)		0.02 (0.267)		0.003 (0.867)	
**Sex**								
Male	Reference	Reference	Reference		Reference	Reference	Reference	
Female	0.26 (< 0.001)	0.22 (< 0.001)	0.05 (0.262)		0.2 (0.003)	0.17 (0.011)	0.03 (0.605)	
**Han Nationality**								
Yes	Reference		Reference		Reference		Reference	Reference
No	-0.20 (0.038)	-0.18 (0.051)	-0.06 (0.293)		-0.14 (0.132)		0.18 (0.032)	0.19 (0.022)
**Residence**								
Urban	Reference		Reference		Reference		Reference	
Rural	-0.14 (0.069)		-0.08 (0.103)		-0.05 (0.533)		-0.01 (0.827)	
**Per capita household income**	0.00001 (< 0.001)	0.00001 (0.001)	0.000004 (0.0498)	0.000004 (0.04)	0.00001 (< 0.001)	0.00001 (0.002)	-0.000002 (0.573)	
**BMIz**	0.09 (0.005)	0.07 (0.036)	-0.04 (0.071)		0.09 (0.004)	0.07 (0.018)	0.06 (0.032)	0.07 (0.02)
**Systolic blood pressure**	0.004 (0.199)		0.0007 (0.722)		0.001 (0.626)		0.003 (0.261)	
**Diastolic blood pressure**	0.005 (0.234)		0.002 (0.49)		0.001 (0.708)		0.003 (0.406)	
**3-day average energy**	0.00001 (0.849)		-0.00002 (0.638)		0.00003 (0.637)		-0.00005 (0.324)	
**3-day average carbohydrat**	-0.0006 (0.126)		-0.0003 (0.172)		-0.0003 (0.463)		-0.0002 (0.495)	
**3-day average fat**	0.002 (0.1)		0.0002 (0.669)		0.001 (0.183)		-0.0009 (0.295)	
**3-day average protein**	0.002 (0.236)		0.0006 (0.537)		0.002 (0.273)		-0.00005 (0.973)	
**Drinking**								
No	Reference	Reference	Reference	Reference	Reference	Reference	Reference	Reference
Yes	-0.36 (0.003)	-0.33 (0.006)	-0.15 (0.049)	-0.16 (0.04)	-0.23 (0.058)	-0.19 (0.098)	-0.15 (0.173)	-0.13 (0.232)

*TC* Total cholesterol, *HDL-C* High density lipoprotein cholesterol, *LDL-C* Low density lipoprotein cholesterol, *TG* Triglyceride

### Propensity score matching

**Fig. 1:**
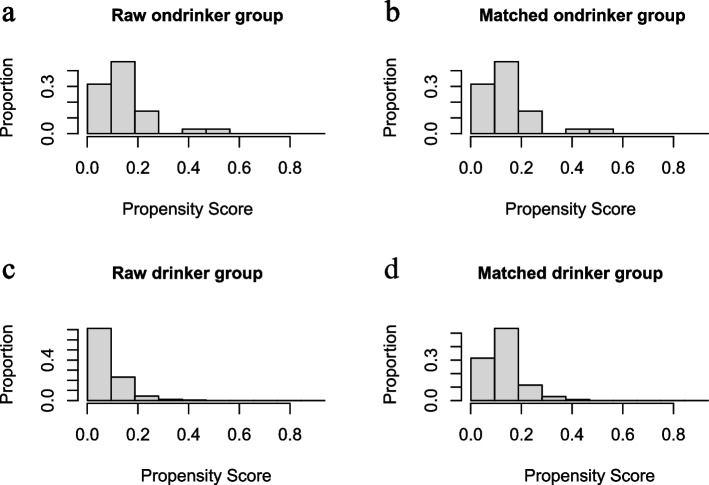
The distribution of propensity scores before and after matching. **a** Nondrinker group before matching, **b** Nondrinker group after matching, **c** Drinker group before matching, **d** Drinker group after matching

To strengthen the conclusions, we used PSM to further analyze the association between alcohol consumption and blood lipids in children and adolescents (35 drinkers and 140 matched nondrinkers). As shown in Fig. 1, the drinkers and nondrinkers groups had better comparability after PSM. Table 3 also indicates that there was no significant difference between the potential confounding factors of the two groups after PSM. PSM analysis indicated that the children and adolescents drinkers had significantly lower TC values (3.8 mmol/L for nondrinkers versus 3.5 mmol/L for drinkers, *p* = 0.002) and HDL-C (1.3 mmol/Lfor nondrinkers versus 1.2 mmol/L for drinkers, *p* = 0.012); however, no significant association was observed between alcohol consumption and LDL-C (2.1 mmol/L for nondrinkers versus 2.0 mmol/L for drinkers, *p* = 0.166) or TG (0.9 mmol/L for nondrinkers versus 0.8 mmol/L for drinkers, *p* = 0.194) levels (Table 3).

**Table 3:** Characteristics of the participants according to their alcohol consumption status after propensity score matching

	**Nondrinker**	**Drinker**	***P* value**
**Participants, n**	140	35	
**Age (years), median (25th-75th percentile)**	15.0 (14.0–16.0)	15.0 (13.0–17.0)	0.753
**Sex, n (%)**			0.900
Male	100 (71.4)	26 (74.3)	
Female	40 (28.6)	9 (25.7)	
**Han Nationality, n (%)**			0.475
Yes	119 (85.0)	32 (91.4)	
No	21 (15.0)	3 (8.6)	
**Residence, n (%)**			0.181
Urban	41 (29.3)	15 (42.9)	
Rural	99 (70.7)	20 (57.1)	
**Per capita household income (RMB), median (25th-75th percentile)**	6102.1 (3139.6–10,848.1)	6650.0 (4252.5–11,976.4)	0.324
**BMIz, median (25th-75th percentile)**	-0.3 (-0.7–0.4)	-0.2 (-0.8–0.4)	0.748
**Blood pressure (mmHg)**			
Systolic blood pressure**,** median (25th-75th percentile)	107.7 (100.0–116.0)	109.3 (100.0–111.7)	0.571
Diastolic blood pressure**,** median (25th-75th percentile)	70.0 (63.8–78.0)	71.3 (67.3–78.3)	0.493
**3-day average dietary value**			
3-day average energy (kcal), median (25th-75th percentile)	2106.6 (1648.0–2530.7)	2349.6 (1961.0–2529.2)	0.146
3-day average carbohydrate (g), mean (SD)	301.7 (95.6)	310.9 (93.9)	0.612
3-day average fat (g), median (25th-75th percentile)	67.5 (46.2–90.8)	79.1 (51.7–99.0)	0.228
3-day average protein (g), median (25th-75th percentile)	63.7 (47.1–81.9)	69.5 (55.7–84.4)	0.305
**Blood lipids (mmol/L), median (25th-75th percentile)**			
Total cholesterol	3.8 (3.4–4.3)	3.5 (3.1–3.8)	0.002
High density lipoprotein cholesterol	1.3 (1.1–1.6)	1.2 (1.0–1.4)	0.012
Low density lipoprotein cholesterol	2.1 (1.7–2.6)	2.0 (1.8–2.2)	0.166
Triglyceride	0.9 (0.6–1.5)	0.8 (0.6–1.1)	0.194

## Discussion

In this study, we used the CHNS database to investigate the association between alcohol consumption and blood lipids of children and adolescents. The present study was the first that we knew of to specifically investigate this association.

We observed that the children and adolescents who drank alcohol had significantly lower TC and HDL-C levels. However, no significant association was observed between alcohol consumption, and LDL-C and TG. After using multivariate regression analysis to adjust for the confounding factors that significantly affect blood lipids, the conclusion remained the same. We then used the PSM method to further characterize the association between alcohol consumption and blood lipids in order to increase the reliability of our conclusions. Applying PSM in this study reduced the number of samples in the nondrinking group from 373 to 140, and no significant difference in confounding factors was found between nondrinkers and drinkers. And after PSM there were no changes in our conclusions, which showed the credibility of our conclusions.

The results of research on the association between adult drinking and blood lipids remain somewhat controversial, in that some studies [8–10, 24–28] have suggested that drinking increases blood lipids and another [11] suggested that drinking does not affect blood lipids; however, relatively few studies have suggested that drinking decreases blood lipids in adults. A meta-analysis of 25 experimental studies found that an increase in alcohol intake of 30 g/day in adults resulted in a mean HDL-C increase of 8.3% [24]. Some previous studies involving adults also found that alcohol consumption was associated with a higher HDL-C, in a dose–response manner, and a higher TC concentration [25–28]. The mechanism of HDL-C elevation caused by drinking in adults may be related to ethanol promoting HDL-C synthesis in the liver and reducing lipoprotein lipase activity [12]. The conclusion on the association between alcohol consumption and blood lipid in children and adolescents is different from that in adults, which might be attributable to the following factors: First, children and adolescents are in a period of rapid growth and development, and their nutritional intake is more important than that in adults. Ethanol can reduce lipid absorption in the digestive tract [29–32]. This may be more sensitive in children and adolescents, reducing their blood lipids. We found that alcohol consumption in children and adolescents was negatively associated with blood lipids, which suggests that blood lipid reductions were somewhat related to lipid absorption. Second, adults tend to have a longer drinking history and higher total alcohol consumption than children and adolescents. Drinking might affect blood lipids differently during different age stages. Third, drinking alcohol is associated with high levels of steroid hormones, which may increase the effect of steroid hormones on lowering total cholesterol [33]. In the two above-mentioned studies [14, 15] on the association between alcohol consumption and blood lipids, the conclusions were also inconsistent, with one suggesting that there is a significant positive association between alcohol consumption and blood lipids [15], and the other that there was no such association [14]. The difference between these results and ours might be attributable to the study populations, since those two studies included non Chinese adolescents (mainly white, 12–19 years old) while we included Chinese children and adolescents (mainly Han, 7–18 years old).

Considering the research design and sample size in this study, we used multiple methods to correct for the effects of confounding factors. In addition to univariate and multivariate linear regression analyses, we also applied PSM. PSM can be used to reduce confounding biases in retrospective analyses of clinical trial data sets, registries, observational studies, and electronic medical records to improve comparability among groups, and it has been widely used in clinical research [34–36]. As an alternative to multivariate regression analysis, PSM attempts to reduce confounder effects by matching already-treated subjects with control subjects who exhibit a similar propensity for treatment based on preexisting covariates that influence treatment selection. PSM therefore establishes a new control group by excluding control subjects with outlying data. This new control group is less influenced by covariates, allowing for more accurate measurements of the variables [37]. PSM is uniquely valuable in its utility and simplicity, but has the disadvantge of requiring data removal.

Several limitations of the present study should be considered. First, there was no amount and the length of alcohol consumption and standard for mild and heavy drinking in children and adolescents. Therefore, the dose effect association between alcohol consumption and blood lipids cannot be studied, which makes the demonstration intensity of the association between alcohol consumption and blood lipids not strong. More accurate grouping may produce more-reliable information. Second, blood lipids were analyzed as continuous variables in our study because there was no testing standard for the blood lipids of children. If the adult blood lipid test standards were applied, there would be almost no children and adolescents with high blood lipids or dyslipidemia in the population we collected, so blood lipids cannot be analyzed as classified variables in the present study. Third, this is a cross-sectional study which is severely limited in causal inference. A larger sample or prospective study is still needed to further determine the relationship between alcohol consumption and blood lipids in children and adolescents. Fourth, due to the data sourced from CHNS, there is a lack of research factors that may affect blood lipids, such as fish oil intake and familial hypercholesterolemia.

## Conclusions

This study suggests that alcohol consumption has significant negative association with TC and HDL-C in children and adolescents, but not with LDL-C or TG. Future prospective studies are needed to confirm these findings and to determine the health implications, such as for cardiovascular or other diseases.

## Data Availability

Datasets used are available from the website, https://www.cpc.unc.edu/projects/china/data/datasets/data-downloads-registration. Enter this website and fill in several required information (such as email and country), then the data can be freely downloaded.
